# Gene regulatory cascade of senescence-associated NAC transcription factors activated by ETHYLENE-INSENSITIVE2-mediated leaf senescence signalling in *Arabidopsis*

**DOI:** 10.1093/jxb/eru112

**Published:** 2014-03-22

**Authors:** Hyo Jung Kim, Sung Hyun Hong, You Wang Kim, Il Hwan Lee, Ji Hyung Jun, Bong-Kwan Phee, Timilsina Rupak, Hana Jeong, Yeonmi Lee, Byoung Seok Hong, Hong Gil Nam, Hye Ryun Woo, Pyung Ok Lim

**Affiliations:** ^1^Center for Plant Aging Research, Institute for Basic Science (IBS), Daegu 711-873, Republic of Korea; ^2^Department of New Biology, Daegu Gyeongbuk Institute of Science & Technology (DGIST), Daegu 711-873, Republic of Korea; ^3^Department of Life Sciences, POSTECH, Pohang, Gyeongbuk 790-784, Republic of Korea; ^4^School of Interdisciplinary Bioscience and Bioengineering, POSTECH, Pohang, Gyeongbuk 790-784, Republic of Korea

**Keywords:** *Arabidopsis*, EIN2-mediated senescence signalling, EIN3, gene, NAC transcription factor.

## Abstract

Leaf senescence is a finely tuned and genetically programmed degeneration process, which is critical to maximize plant fitness by remobilizing nutrients from senescing leaves to newly developing organs. Leaf senescence is a complex process that is driven by extensive reprogramming of global gene expression in a highly coordinated manner. Understanding how gene regulatory networks involved in controlling leaf senescence are organized and operated is essential to decipher the mechanisms of leaf senescence. It was previously reported that the trifurcate feed-forward pathway involving *EIN2*, *ORE1*, and *miR164* in *Arabidopsis* regulates age-dependent leaf senescence and cell death. Here, new components of this pathway have been identified, which enhances knowledge of the gene regulatory networks governing leaf senescence. Comparative gene expression analysis revealed six senescence-associated NAC transcription factors (TFs) (ANAC019, AtNAP, ANAC047, ANAC055, ORS1, and ORE1) as candidate downstream components of ETHYLENE-INSENSITIVE2 (EIN2). EIN3, a downstream signalling molecule of EIN2, directly bound the *ORE1* and *AtNAP* promoters and induced their transcription. This suggests that EIN3 positively regulates leaf senescence by activating *ORE1* and *AtNAP*, previously reported as key regulators of leaf senescence. Genetic and gene expression analyses in the *ore1 atnap* double mutant revealed that ORE1 and AtNAP act in distinct and overlapping signalling pathways. Transient transactivation assays further demonstrated that ORE1 and AtNAP could activate common as well as differential NAC TF targets. Collectively, the data provide insight into an EIN2-mediated senescence signalling pathway that coordinates global gene expression during leaf senescence via a gene regulatory network involving EIN3 and senescence-associated NAC TFs.

## Summary

The EIN2-mediated senescence signalling pathway coordinates the expression of genes during leaf senescence via the gene regulatory network involving EIN3 and senescence-associated NAC TFs.

## Introduction

Leaf senescence is a well-orchestrated and genetically programmed cell death process that constitutes the final stage of leaf development (Lim *et al.*, 2007). During leaf senescence, cells in a leaf undergo a dramatic transition in cellular metabolism and the degradation of cellular structures in an orderly manner, resulting in the recycling of nutrients to newly developing vegetative and reproductive organs (Nooden, 1988; Nam, 1997; Lim *et al.*, 2007). Leaf senescence proceeds with the age of a leaf; however, it is also influenced by various endogenous factors, including phytohormones, and external environmental factors, such as salt stress, extreme temperatures, and pathogen attack (Thomas and Stoddart, 1980; Weaver *et al.*, 1998; Dai *et al.*, 1999; Quirino *et al.*, 2000; Buchanan-Wollaston *et al.*, 2005; Lim *et al.*, 2007). Thus, leaf senescence is a very complicated process incorporating multiple developmental and environmental signals, which involves extensive reprogramming and modulation of gene expression.

Intensive genetic and genomic studies during the past decade have led to major advances in our understanding of leaf senescence at the molecular level. In particular, recent genome-wide transcriptome studies have uncovered a global picture of the leaf senescence process, which involves thousands of senescence-associated genes (SAGs) that are differentially expressed during leaf senescence (Buchanan-Wollaston *et al.*, 2005; van der Graaff *et al.*, 2006; Breeze *et al.*, 2011). Given that the expression of >200 transcription factor (TF) genes is altered during leaf senescence (Buchanan-Wollaston *et al.*, 2003; Balazadeh *et al.*, 2008; Liu *et al.*, 2011; Li *et al.*, 2012; Guo, 2013) and TFs regulate the transcription of their target genes in a spatiotemporal-specific manner, gene regulatory networks composed of interactions between these TFs and their targets have been implicated in controlling leaf senescence. Indeed, by taking advantage of high-throughput and computational analyses, researchers have begun to identify gene regulatory networks involved in the leaf senescence process. For example, a gene regulatory network model has been reconstructed using selected SAGs from microarray-based temporal expression profiling during *Arabidopsis* leaf senescence (Breeze *et al.*, 2011). The proposed network model predicts the effects of a plant-specific NAC (NAM/ATAF1,2/CUC2) TF, ORESARA1 (ORE1/NAC2/ANAC092), which is known to be one of the central positive regulators of leaf senescence, on the expression of multiple known downstream target genes and several stress-related TFs. Hickman *et al.* (2013) proposed a gene regulatory network model involving ANAC019, ANAC055, and ANAC072, based on high-throughput yeast one-hybrid (Y1H) assays and time-course gene expression data. Although initial attempts have been made to characterize gene regulatory networks important for leaf senescence, what the gene regulatory network involving TFs important for the control of leaf senescence is composed of and how it is operated have been largely unexplored.

It was previously reported that the trifurcate feed-forward pathway, which involves ETHYLENE-INSENSITIVE2 (EIN2/ORE2/ORE3), *miRNA164* (*miR164*), and ORE1, regulates age-dependent leaf senescence and cell death (Kim *et al.*, 2009). EIN2, a central signalling component required for ethylene responses (Alonso *et al.*, 1999), induces *ORE1* in an age-dependent manner. *ORE1* is negatively regulated by *miR164* in young leaves, which is relieved in old leaves due to the age-dependent down-regulation of *miR164* by EIN2. In young *Arabidopsis* leaves, *miR164* suppresses *ORE1*, which positively regulates leaf senescence. However, in old leaves, EIN2 suppresses *miR164* and thereby induces *ORE1* expression, which leads to leaf senescence. Based on the results of mathematical modelling and genetic analyses with the *ein2* and *ore1* mutants, it was further suggested that EIN2 utilizes another pathway that does not include ORE1. Recently, EIN3, a well-known key TF in the EIN2-mediated ethylene signalling cascade (Chao *et al.*, 1997), has been shown to be involved in the trifurcate feed-forward pathway of age-dependent senescence and cell death (Li *et al.*, 2013). EIN3 induces the accumulation of *ORE1* transcript in an age-dependent manner by directly repressing *miR164* transcription. However, how EIN2-mediated senescence signalling is transduced to ORE1 and how a gene regulatory network involving ORE1 is organized to regulate leaf senescence have not been investigated.

In this study, novel components in the trifurcate feed-forward pathway were identified and characterized to augment understanding of the composition, organization, and function of the gene regulatory networks that govern leaf senescence. As a first step toward expanding the trifurcate feed-forward pathway for leaf senescence, six senescence-associated NAC TFs were identified, including ORE1 and AtNAP/ANAC029, as candidate downstream targets of EIN2, and whether these NAC TF genes were acting downstream of EIN3 was further examined. Y1H and chromatin immunoprecipitation (ChIP) assays demonstrated that EIN3 directly bound to the promoters of the *ORE1* and *AtNAP* genes. Transiently overexpressed *EIN3* in *Arabidopsis* protoplasts was sufficient to activate the expression of *ORE1* and *AtNAP*. Genetic and gene expression analyses in *ore1 atnap* double mutants revealed that ORE1 and AtNAP have partially additive functions in age-dependent and artificially induced leaf senescence. Using transient transactivation assays, it was further found that ORE1 and AtNAP regulate common as well as distinct NAC TF targets. Based on these data, a plausible model for an EIN2–EIN3–NAC TF gene regulatory cascade that has an important role in the control of leaf senescence is proposed. Collectively, the data provide insight into how the EIN2-mediated senescence signalling pathway coordinates global gene expression during leaf senescence via a gene regulatory network involving EIN3 and NAC TFs.

## Materials and methods

### Plant materials and growth conditions

*Arabidopsis thaliana* ecotype Columbia (Col) is the parent strain for all mutants used in this study. The *ore1-2*, *ore1-3*, *ein2-34/ore3-1*, *ore9-1*, and *ore12-1* mutants were described previously (Woo *et al.*, 2001; Kim *et al.*, 2006; Kim *et al.*, 2009). The *ein3-1eil1-1* (*ein3-like 1-1*) (Alonso *et al.*, 2003) mutant and the *ein3 eil1 ebf1* (*ein3-binding f-box1*) *ebf2* mutant containing estradiol-inducible *EIN3-3XFLAG* (*iE/qm*) (An *et al.*, 2010) were kindly provided by H. Guo (Peking University, China). The *35S promoter (35Sp*)*:EIN3-FLAG* mutant was kindly provided by S.D. Yoo (Korea University, South Korea). The *atnap* T-DNA insertion line (SALK_005010C) was obtained from the Salk T-DNA insertion collection (Alonso *et al.*, 2003). The genotype of each line was confirmed by PCR-based genotyping (Supplementary Table S1 available at *JXB* online). The *35Sp:EIN3-FLAG ore1-2* and *35Sp:EIN3-FLAG atnap* were generated by genetic cross, and double homozygous lines were identified through PCR-based genotyping (Supplementary Table S1). Plants for physiological experiments were grown in an environmentally controlled growth room at 22 °C with 16h of light from a fluorescent light at 100 μmol m^–2^ s^–1^.

### Assays of leaf senescence

Age-dependent leaf senescence was assayed as described by Woo *et al.* (2001). The photochemical efficiency of photosystem II (PSII) was deduced from the chlorophyll fluorescence (Oh *et al.*, 1996) using an Imaging-PAM chlorophyll fluorometer (Heinz Walz GmbH, Germany). The ratio of the maximum variable fluorescence to the maximum yield of fluorescence, which corresponds to the potential quantum yield of the photochemical reactions of PSII, was used as a measure of the photochemical efficiency of PSII (John *et al.*, 1995; Raggi, 1995; Oh *et al.*, 1997). Chlorophyll was extracted from individual leaves by heating in 95% ethanol at 80 °C. The chlorophyll concentration per fresh weight of leaf tissue was calculated as described by Lichtenthaler (1987). For dark-induced leaf senescence experiments, the third or fourth rosette leaves of wild-type or mutant plants at 12 d of leaf age were carefully detached and incubated in 3mM MES buffer (pH 5.7) at 22 °C in the dark for the designated time. For hormone treatment, leaves were floated on 3mM MES buffer (pH 5.7) in the presence or absence of 50 μM 1-aminocyclopropane-1-carboxylic acid (ACC; Sigma-Aldrich, USA) or 50 μM methyl jasmonate (MeJA; Sigma-Aldrich, USA) for 5 d. All hormonal treatments were performed at 22 °C under continuous light.

### RNA isolation and quantitative reverse transcription–PCR (qRT–PCR)

Total RNA was isolated from the third and fourth rosette leaves using WelPrep total RNA isolation reagent (WELGENE, Republic of Korea), according to the manufacturer’s instructions. First-strand cDNA was synthesized from 1.0 μg of RNA using the ImProm II Reverse Transcriptase system kit (Promega, USA), followed by quantitative PCR (qPCR) analysis to determine gene expression levels (Bio-Rad, CFX96 Touch Real-Time PCR Detection System, USA). Primers used for qRT–PCR are listed in Supplementary Table S1 at *JXB* online. Transcript levels were calculated using the comparative threshold (C_T_) method, with *ACT2* (At3g18780) as the internal control.

### Yeast one-hybrid (Y1H) assays

The DupLEX-A system (OriGene Technologies, USA) was used with slight modifications for Y1H analysis of gene interactions. *EIN3* full-length cDNA was cloned into the pJG4-5 prey vector, which includes a B42 transcriptional activation domain. Approximately 2kb of the *ORE1* and *AtNAP* promoters were cloned separately into the *lacZ* (*β-galactosidase*) reporter plasmid pSH18-34. The yeast strain EGY48 (*MATa*, *trp1*, *his3*, *ura3*, *leu2*::6 LexAop-*LEU2*) was transformed with the indicated combinations of plasmids. Interactions were tested on 5-bromo-4-chloro-3-indolyl-β-d-galactopyranoside (X-*gal*) medium (Ryu *et al.*, 2005).

### Transient expression assay in *Arabidopsis* protoplasts

For *luciferase* (*LUC*) reporter constructs, the promoters of *ORE1*, *AtNAP*, *ANAC003*, *ANAC041*, *ANAC079*, *VND-INTERACTING2* (*VNI2*)/*ANAC083*, *ANAC087*, and *ANAC102* were amplified from genomic DNA, cloned into *pCR-CCD F* (Kim and Somers, 2010), and recombined into the gateway version of the *pGreen0800-LUC* vector (Hellens *et al.*, 2005), which contains *35Sp:RLuc* (Renilla *luciferase*) as an internal control. *Arabidopsi*s protoplasts were isolated and transfected as described (Hwang and Sheen, 2001; Yoo *et al.*, 2007). Transfected protoplasts were incubated for 6h at 22 °C under dim light (5 μE m^–2^ s^–1^) and the luciferase activity was measured using the Dual-Luciferase reporter assay system (Promega, USA), according to the manufacturer’s instructions.

### Protein isolation and western blotting

To induce the expression of *EIN3-FLAG*, the 3- and 5-week-old *iE/qm* transgenic plants were sprayed with 20 μM and 100 μM estradiol for 6h, respectively. The third and fourth leaves were harvested, ground in liquid N_2_, and lysed with extraction buffer containing 50mM Tris-HCl (pH 7.5), 150mM NaCl, 10mM EDTA, 0.1% Nonidet P-40, 50 μM MG132, 1mM phenylmethylsulphonyl fluoride (PMSF), and a protease inhibitor cocktail. The protein extracts were heated at 95 °C for 5min in SDS–PAGE sample loading buffer, separated on 10% SDS–polyacrylamide gels, and transferred to polyvinylidene fluoride (PVDF) membranes (Gamble *et al.*, 2002). The blot was probed with a monoclonal anti-FLAG antibody (Sigma-Aldrich, USA).

### Chromatin immunoprecipitation (ChIP)-qPCR

Third and fourth leaves from 5-week-old *iE/qm* transgenic plants treated with 100 μM estradiol for 6h were harvested, and 2g was fixed in 1% formaldehyde solution and cross-linked under vacuum for 15min. A final concentration of 0.25M glycine was used to quench the formaldehyde for 5min under vacuum. After washing twice with cold deionized water, the tissue was ground in liquid N_2_ and extraction of chromatin was performed as described by Zhu *et al.* (2012). Prior to immunoprecipitation, 5 μg of anti-FLAG monoclonal antibody (Sigma-Aldrich, USA) was pre-incubated with 20 μl of protein A+G magnetic beads (Millipore, USA) at 4 °C on a rotator overnight. Sonicated chromatin supernatant (250 μl) was diluted to 500 μl and pre-cleared with 20 μl of protein A+G magnetic beads for 1h at 4 °C. Supernatants were incubated with the prepared antibody-bound beads at 4 °C for 2h, and beads were washed sequentially with low-salt wash buffer, high-salt wash buffer, and TE buffer. Elution and reverse cross-linking was performed as previously described (Zhu *et al.*, 2012). The resulting immunoprecipitated DNA was purified with the Qiaquick PCR purification kit (Qiagen, USA), and used for qPCR to examine the enrichment of target genes using the primers listed in Supplementary Table S1 at *JXB* online.

## Results

### Identification of the senescence-associated NAC TFs acting downstream of EIN2

In an effort to expand the trifurcate pathway for leaf senescence, the aim was to identify molecular components functioning downstream of EIN2 in the regulation of leaf senescence. Genes whose expression is changed during leaf senescence and altered in the *ein2* mutant would be good candidates as potential downstream targets of EIN2. NAC TF family proteins were the top candidates because publicly available microarray data revealed that NAC TF family genes are significantly up-regulated during leaf senescence in *Arabidopsi*s, and the expression of some NAC TFs is altered in the *ein2* mutant during leaf senescence (Schmid *et al.*, 2005; Kim *et al.*, 2009; Asahina *et al.*, 2011; Breeze *et al.*, 2011).

**Fig. 1.**
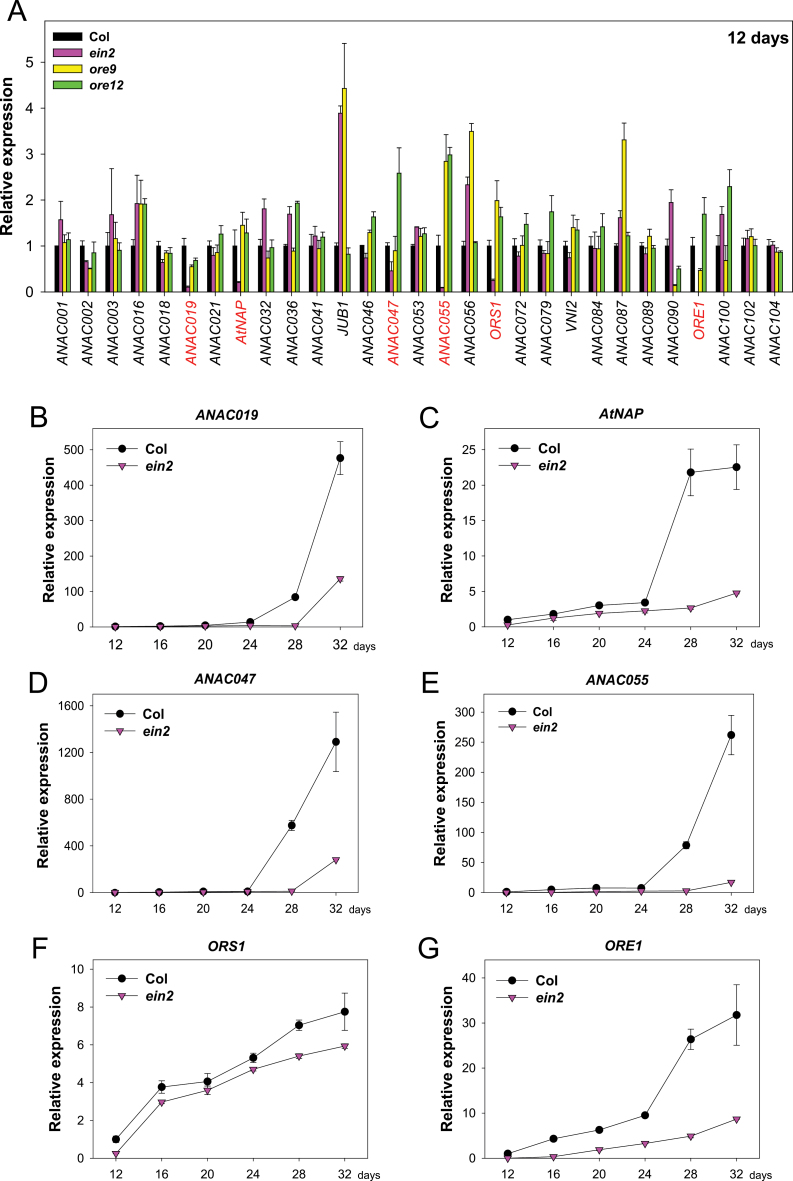
Identification of the six senescence-associated NAC TFs as potential downstream components of EIN2. (A) Expression of 29 senescence-associated NAC TF genes in Col, *ein2*, *ore9*, and *ore12* mutant leaves at the mature stage (12-day-old third and fourth rosette leaves). Transcript levels of each TF gene were examined by qRT–PCR. For qRT–PCR, *ACT2* was used as an internal control. Transcript abundance of the NAC TF genes in each mutant was determined relative to that in wild-type leaves. The error bars represent the standard deviation (SD; *n*=4). The six NAC TF genes whose expression was decreased by >50% in the mature leaves of *ein2* mutants compared with the wild-type are highlighted by grey text. (B–G) Age-dependent changes in the expression of candidate NAC TF genes downstream of EIN2. Transcript levels of *ANAC019* (B), *AtNAP* (C), *ANAC047* (D), *ANAC055* (E), *ORS1* (F), and *ORE1* (G) were analysed by qRT–PCR in the third and fourth rosette leaves from wild-type and *ein2* plants at the indicated ages. Transcript levels of each gene during leaf ageing were determined relative to levels in wild-type 12-day-old leaves. Error bars represent the SD (*n*=4).

The focus of this strudy was the 29 NAC TF genes whose expression is increased at least 5-fold in senescent leaves, based on data from AtGenExpress (Schmid *et al.*, 2005). The expression of the 29 senescence-induced NAC TF genes was examined in wild-type (Col) and *ein2*/*ore3* mutant leaves at the mature stage (12-day-old third and fourth rosette leaves) by qRT–PCR (Fig. 1A). The expression analysis was also performed in the *ore9* and *ore12* mutants, which are well-known delayed leaf senescence mutants (Woo *et al.*, 2001; Kim *et al.*, 2006). Comparative expression analysis in these three mutants would allow the candidate downstream targets of EIN2 to be narrowed down by eliminating the NAC TFs whose expression is preferentially affected by delayed leaf senescence itself. As expected, the expression of *ORE1* was strongly reduced in the mature leaves of *ein2* (Fig. 1A). In addition to *ORE1*, *ANAC019*, *AtNAP*, *ANAC047*, *ANAC055*, and *ORE1 SISTER1* (*ORS1*)/*ANAC059* transcripts were significantly decreased in the mature leaves of *ein2*, compared with the reduction in the *ore9* and *ore12* mutants, implying that these six NAC TF genes are potential downstream targets of EIN2 in the control of the leaf senescence process.

Expression changes in the six NAC TF genes in wild-type and *ein2* mutant leaves were further compared at 4 d intervals during leaf ageing (Fig. 1B–G). Consistent with previous findings (Schmid *et al.*, 2005), the expression of the six NAC TF genes increases as a leaf gets older in wild-type leaves. Overall, these NAC TF genes were expressed at lower levels in *ein2* leaves compared with wild-type leaves throughout the developmental stages of the leaf examined. The expression kinetics of each NAC TF gene in the *ein2* mutant, however, were distinct during leaf ageing. For example, the abundance of *ANAC019* and *ANAC047* transcripts was dramatically increased in 28-day-old wild-type leaves, but transcripts of these genes in the *ein2* leaves reached similar levels to those in 28-day-old wild-type leaves ~4 d later (Fig. 1B, D). In the case of *AtNAP*, *ANAC055*, and *ORE1*, transcript levels were also strongly induced in 28-day-old wild-type leaves, but did not significantly change in the *ein2* mutant leaves until 32 d (Fig. 1C, E, G). These data indicate that *ANAC019*, *AtNAP*, *ANAC047*, *ANAC055*, and *ORE1* are preferentially under the control of EIN2 during leaf ageing. In contrast, the expression kinetics of *ORS1* during leaf ageing were similar in the wild-type and *ein2* mutants, although its expression was lower in the *ein2* leaves at all ages examined (Fig. 1F). This implies that *ORS1* expression might be controlled by EIN2-independent as well as EIN2-dependent senescence signals. Collectively, these results suggest that at least six NAC TFs, including ORE1, act downstream of EIN2, which may be new components in the gene regulatory network governed by EIN2-mediated senescence signals.

### EIN3 promotes leaf senescence through the activation of the two master NAC TFs, ORE1 and AtNAP

Next, experiments were carried out to examine how the EIN2-mediated senescence signal is transferred into the six NAC TFs. EIN3 has been long known as a key TF in EIN2-mediated ethylene signalling (Chao *et al.*, 1997). Recent evidence indicates that EIN3 might function as an upstream regulator of these NAC TF genes during leaf senescence. First, *EIN3* expression is induced durng leaf ageing, and the double mutant of *EIN3* and its close homologue *EIL1*, *ein3 eil1*, exhibits delayed age-dependent leaf senescence (Li *et al.*, 2013). Secondly, a previous ChIP-seq analysis revealed that EIN3 binds to the promoter of *ORE1* after ethylene treatment in *Arabidopsis* seedlings (Chang *et al.*, 2013). Thus, it was investigated whether EIN3 indeed acts upstream of the NAC TF genes whose expression was preferentially controlled by EIN2.

**Fig. 2.**
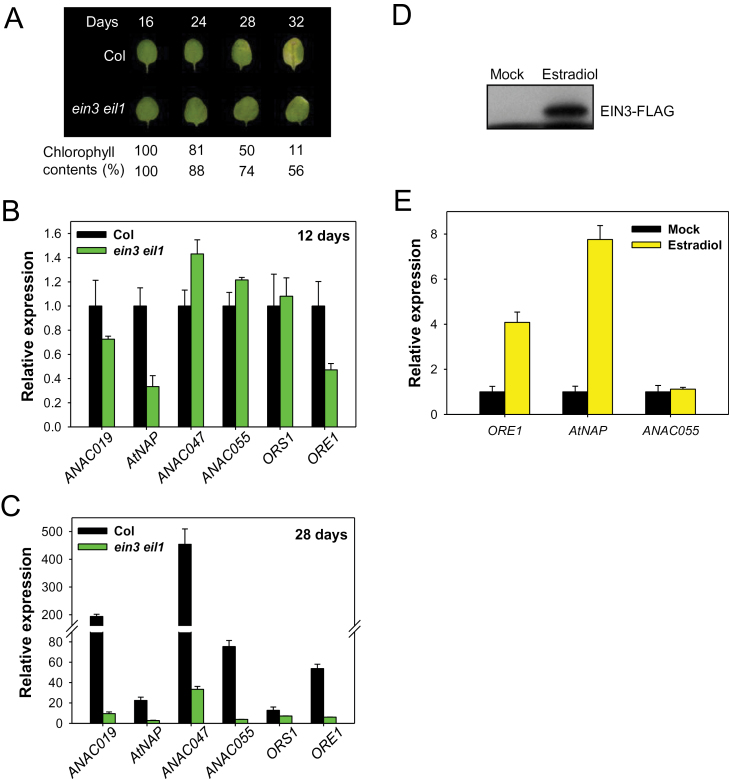
*EIN3* is necessary and sufficient to induce the expression of the *ORE1* and *AtNAP* genes. (A) Changes in chlorophyll content in the third and fourth rosette leaves of Col and *ein3 eil1* mutant plants during leaf ageing. The photographs show representative leaves at the indicated age. Chlorophyll content is compared with the values from each genotype at day 12. (B and C) Expression of the six *NAC* TF genes in the third and fourth leaves of wild-type and *ein3 eil1* mutants at 12 d (B) and 28 d (C) of leaf age. For qRT–PCR, *ACT2* was used as an internal control. Transcript levels of each gene were determined relative to levels in wild-type 12-day-old leaves. Error bars represent the SD (*n*=4). (D) Expression of *ORE1*, *AtNAP*, and *ANAC055* in transgenic *ein3 eil1 ebf1 ebf2* plants overexpressing estradiol-inducible *EIN3* (*iE/qm*). Three-week-old *iE/qm* transgenic plants were treated with 20 μM estradiol for 6h, and protein and RNA were isolated from the third and fourth leaves. The tagged EIN3 protein was visualized by immunoblot analysis using an anti-FLAG antibody. (E) For qRT–PCR, *ACT2* was used as an internal control. Transcript levels of each gene after estradiol treatment were determined relative to the mock treatment. The error bars represent the SD (*n*=4).

The effects of the *ein3* mutation on the expression of the six *NAC* TF genes (*ANAC019*, *AtNAP*, *ANAC047*, *ANAC055*, *ORS1*, and *ORE1*) were first monitored (Fig. 2A–C). The *ein3 eil1* double mutant was used instead of the *ein3* single mutant due to the functional redundancy of EIN3 and EIL1 (Alonso *et al.*, 2003; Binder *et al.*, 2004). In mature (12-day-old) leaves, only *ORE1* and *AtNAP* were down-regulated by >50% in the *ein3 eil1* mutant compared with the wild type (Fig. 2B). In contrast, in 28-day-old leaves, all six NAC TF genes were expressed at significantly lower levels in the *ein3 eil1* mutant, probably because of the delayed senescence phenotype of the *ein3 eil1* mutant (Fig. 2A, C). These results imply that EIN3 might play a positive role in controlling leaf senescence through the activation of *ORE1* and *AtNAP.*

The expression of the *ORE1* and *AtNAP* genes was then examined in transgenic plants expressing estradiol-inducible *EIN3-FLAG* in the *ein3 eil1 ebf1 ebf2* quadruple mutant background (*iE/qm*). Three-week-old *iE/qm* plants were sprayed with 20 μM estradiol and the third and fourth leaves were harvested 6h after treatment. As shown in Fig. 2D, *EIN3* protein efficiently accumulated in the *iE/qm* transgenic plants following estradiol treatment. *ORE1* and *AtNAP* transcripts were significantly induced in the *iE/qm* transgenic plants by estradiol treatment, while *ANAC055* transcript did not change (Fig. 2E). Overall, gene expression analysis of the NAC TF genes in the mutant and EIN3-inducible lines demonstrate that EIN3 is necessary and sufficient to induce the expression of *ORE1* and *AtNAP* to promote leaf senescence.

**Fig. 3.**
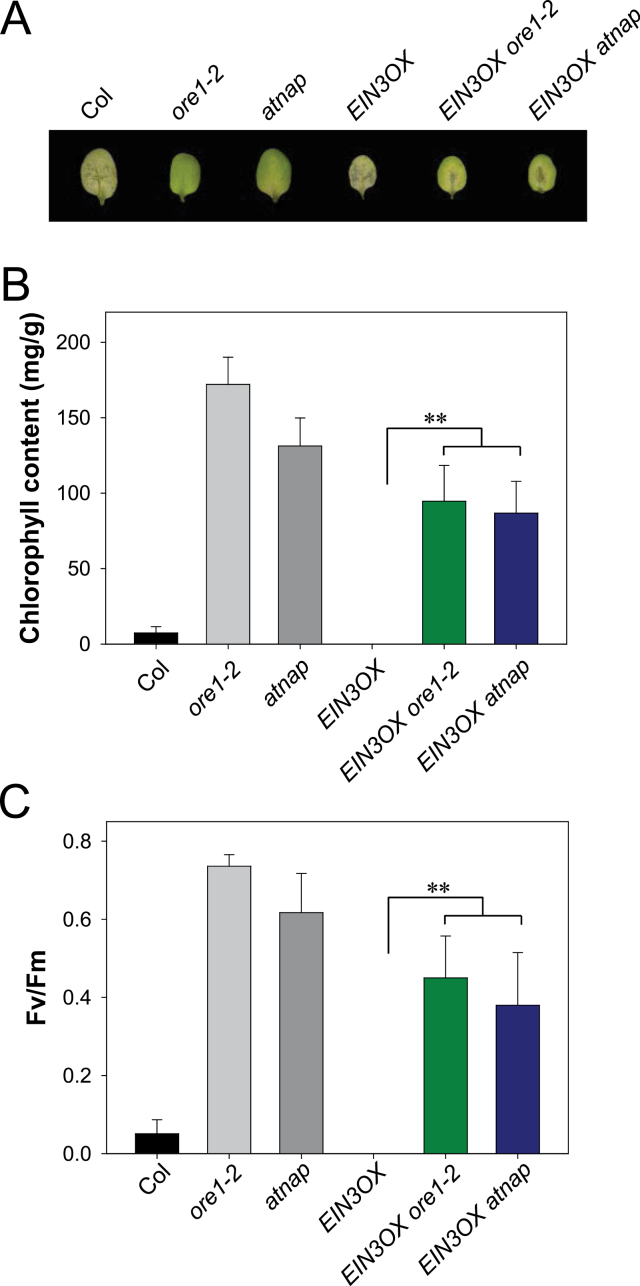
The *ORE1* or *AtNAP* loss-of-function mutant suppresses the early senescence phenotypes of an *EIN3* overexpressor during dark-induced leaf senescence. (A) Representative leaves of Col, *ore1, atnap, EIN3OX*, *EIN3OX ore1*, and *EIN3OX atnap* plants after incubation in darkness for 6 d. (B and C) Analysis of chlorophyll content (B) and photochemical efficiency of PSII (C) of detached leaves of the indicated genotypes at 6 d after dark incubation (Student’s *t*-test, **P<0.01). Error bars indicate the SD (*n* >20).

It is well known that ORE1 and AtNAP are master positive regulators of leaf senescence (Guo and Gan, 2006; Kim *et al.*, 2009). Given that ORE1 and AtNAP function downstream of EIN3 in leaf senescence (Fig. 2), *ORE1* and *AtNAP* loss-of-function mutations would be expected to repress EIN3-induced early senescence. A transgenic line expressing *EIN3-FLAG* driven from the *35S promoter* (*EIN3OX*) was crossed with the *ore1* or *atnap* mutants, and the dark-induced leaf senescence phenotype was examined in the double homozygous lines (Fig. 3). As expected, *EIN3OX* leaves exhibited early senescence phenotypes during dark incubation (Fig. 3). Mutation of *ORE1* partially suppressed the EIN3-induced early leaf senescence phenotype (Fig. 3A–C). Similarly, the loss of chlorophyll content and photochemical efficiency during dark-induced leaf senescence was also delayed in both the *atnap* and *EIN3OX atnap* mutants, compared with wild-type and *EIN3OX* mutant leaves (Fig. 3A–C). The data demonstrated that EIN3 requires both ORE1 and AtNAP to induce leaf senescence. Taken together, these results revealed that EIN2-mediated senescence signalling induced the expression of the *ORE1* and *AtNAP* genes through EIN3, and that this gene regulatory network played a major role in controlling leaf senescence.

### EIN3 activates the expression of the *ORE1* and *AtNAP* genes by directly binding to their promoters

**Fig. 4.**
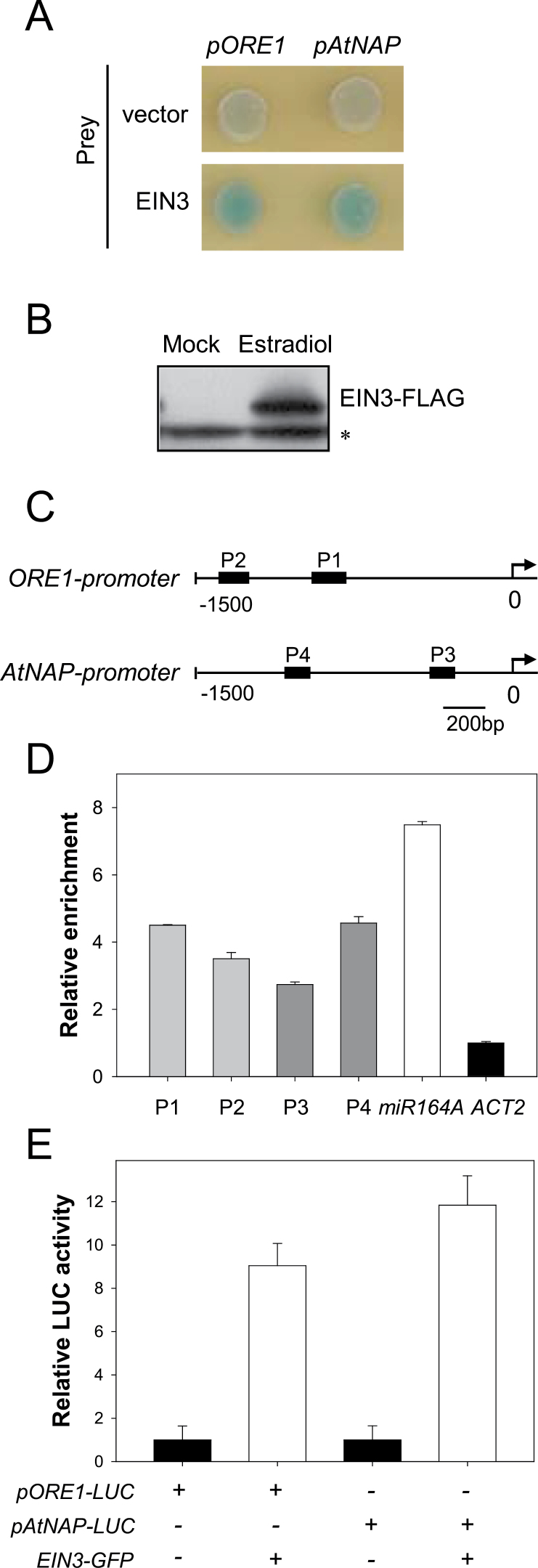
EIN3 binds to the promoters of *ORE1* and *AtNAP*, and induces their transcription. (A) Binding of EIN3 to the promoters of *ORE1* and *AtNAP* in Y1H assay. An effector plasmid containing *EIN3* and a reporter plasmid (*pORE1-lacZ* or *pAtNAP-lacZ*) were co-transformed into the EGY48 yeast strain. The growth of a blue yeast colony on selective medium containing X-*gal* indicates a positive interaction. The effector plasmid without EIN3 (vector alone) plus the reporter plasmid served as a negative control. (B) Protein levels of EIN3 in 5-week-old *iE/qm* transgenic plants treated or not with 100 μM estradiol for 6h. The tagged EIN3 protein was visualized by immunoblot analysis using an anti-FLAG antibody. An asterisk indicates non-specific bands detected by the anti-FLAG antibody. (C) Schematic diagram of the *ORE1* and *AtNAP* promoters. P1–P4 represent the positions of amplicons used for ChIP-qPCR analysis. P1–P4 were chosen because these regions contain putative EIN3 binding sites (EBS, TACAT or TTCAAA). (D) Enrichment of EIN3-associated fragments after ChIP-qPCR. Chromatin from the leaves of 5-week-old *iE/qm* transgenic plants treated with 100 μM estradiol for 6h was immunoprecipitated with an anti-FLAG antibody. Enrichment was quantified by qPCR using specific primers. Fold changes in enrichment were normalized to *ACT2*. The promoter of *miR164A* was used as a positive control. The error bars represent the SD (*n*=4). (E) Transactivation of the *ORE1* and *AtNAP* promoters by EIN3 in *Arabidopsis* protoplasts. Protoplasts were co-transfected with the *pORE1-LUC* or *pAtNAP-LUC* reporter and an effector plasmid expressing EIN3–GFP (green fluorescent protein). Luciferase activity was determined relative to that in protoplasts that were transfected with the reporter plasmid and an effector plasmid expressing GFP only. Relative expression of *ORE1-LUC* or *AtNAP-LUC* was normalized to that of *35Sp:RLuc* (internal control). Error bars represent the SD (*n*=8).

To examine whether the EIN3-mediated activation of the *ORE1* and *AtNAP* genes is achieved by direct binding of EIN3 to their promoters, Y1H analysis was performed (Fig. 4A). The EGY48 yeast strain was co-transfected with an effector plasmid containing the full-length cDNA of *EIN3* fused to the B42 transcriptional activation domain and a reporter vector containing either the *ORE1* or the *AtNAP* promoter fused to the *lacZ* gene. Co-expression of EIN3 induced the expression of the *lacZ* reporter gene driven by the *ORE1* or *AtNAP* promoter (Fig. 4A), indicating that EIN3 bound directly to the promoters of *ORE1* and *AtNAP* in yeast.

ChIP-qPCR using *iE/qm* transgenic plants was carried out to determine whether EIN3 binds to the promoters of the *ORE1* or *AtNAP* genes in plant cells. EIN3-FLAG protein strongly accumulated in the leaves of 5-week-old *iE/qm* transgenic plants treated with 100 μM estradiol for 6h (Fig. 4B). The *miR164A* promoter, which was shown to be a direct target of EIN3 (Li *et al.*, 2013), was greatly enriched when a FLAG antibody was used to immunoprecipitate the FLAG-tagged EIN3 protein. In the same plants, a significant enrichment of EIN3 was also observed in the promoter regions of *ORE1* (P1 and P2) and *AtNAP* (P3 and P4) (Fig. 4C, D), supporting the conclusion that *ORE1* and *AtNAP* are direct downstream targets of EIN3 *in vivo*.

To investigate further whether EIN3 functions as a transcriptional activator of *ORE1* and *AtNAP* in plant cells, luciferase-based transactivation assays were performed using *Arabidopsis* mesophyll protoplasts. A reporter construct containing the firefly *luciferase* (*LUC*) reporter gene under the control of either the *ORE1* or the *AtNAP* promoter (*pORE1-LUC* or *pAtNAP-LUC*) was transfected into protoplasts with or without the *35Sp:EIN3-GFP* effector plasmid. Luciferase activity was significantly increased when either the *pORE1-LUC* or the *pAtNAP-LUC* reporter construct was co-transfected with *35Sp:EIN3-GFP*, compared with controls that were solely transfected with the reporter constructs (Fig. 4E), indicating that EIN3 transactivates the promoters of *ORE1* and *AtNAP* in protoplasts. Taken together, these results demonstrate that EIN3 directly activates *ORE1* and *AtNAP* transcription by binding to their promoter regions.

### ORE1 and AtNAP have partially additive functions in regulating age-dependent and artificially induced leaf senescence

**Fig. 5.**
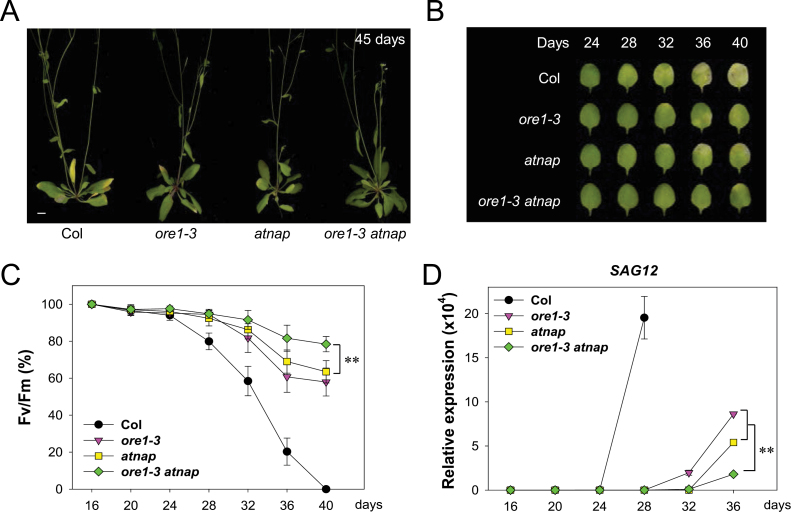
The *ore1 atnap* double mutant exhibited a stronger delay in age-dependent leaf senescence than either mutant alone. (A) Whole-plant phenotypes of Col, *ore1*, *atnap*, and *ore1 atnap* mutant plants at 45 d after germination. The scale bar represents 1cm. (B) Age-dependent senescence phenotype of the third and fourth rosette leaves of Col, *ore1*, *atnap*, and *ore1 atnap* mutant plants at different ages. (C) The photochemical efficiency (*F*_v_/*F*_m_) of PSII was measured from the third and fourth leaves starting at 16 d of leaf age (Student’s *t*-test, **P<0.01). Error bars indicate the SD (*n*=12). (D) Age-dependent changes in *SAG12* gene expression by qRT–PCR analysis. *ACT2* was used as an internal control for qRT–PCR. The transcript level of *SAG12* in wild-type at 12-day-old leaves was set at 1 (Student’s *t*-test, **P<0.01). The error bars represent the SD (*n*=4).

The genetic relationship between *ORE1* and *AtNAP* in leaf senescence was explored by generating an *ore1 atnap* double mutant and analysing the leaf senescence phenotype during leaf ageing. Leaf senescence symptoms were first examined in detail during age-dependent *in planta* senescence. As previously reported (Guo and Gan, 2006; Kim *et al.*, 2009), delayed loss of chlorophyll content with leaf ageing was observed in the *ore1* and *atnap* single mutants (Fig. 5A, B). The 36-day-old leaves from wild-type plants lost 79.7% of the initial photochemical efficiency (*F*_v_/F_m_) of PSII, while leaves from the *ore1* and *atna*p mutants lost 39.3% and 31.0% of their initial PSII activity, respectively (Fig. 5C). Notably, 81.4% of the photochemical efficiency of PSII was retained in the leaves of *ore1 atnap* double mutant leaves at the same age. The expression of *SAG12* also increased dramatically in 28-day-old wild-type leaves, but remained at a very low level until 28 d and 32 d in the *ore1* and *atnap* single mutant leaves, respectively (Fig. 5D). In the *ore1 atnap* double mutant, induction of *SAG12* was delayed even longer than in either of the single mutants (Fig. 5D).

**Fig. 6.**
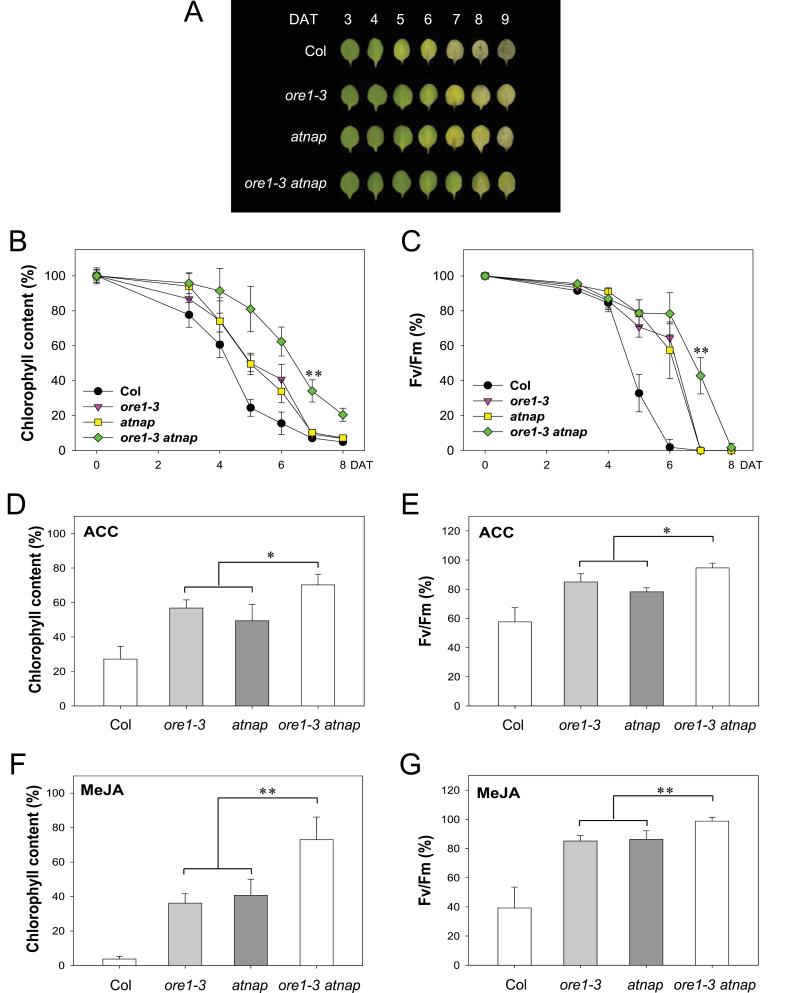
ORE1 and AtNAP play partially additive roles in regulating artificially induced leaf senescence. (A) Phenotypes of Col, *ore1*, *atnap*, and *ore1 atnap* leaves after dark incubation for the indicated times. DAT, days after treatment. (B and C) Changes in photochemical efficiency (*F*_v_/*F*_m_) of PSII (B) and chlorophyll content (C) during dark-induced leaf senescence. Levels of photochemical efficiency and chlorophyll content on the days indicated were determined relative to those before dark incubation (Student’s *t*-test, **P<0.01). Error bars indicate the SD (*n*=6). (D and E) Changes in the chlorophyll content (D) and photochemical efficiency of PSII (E) during ACC-induced leaf senescence. (F and G) Changes in the chlorophyll content (F) and photochemical efficiency of PSII (G) during MeJA-induced leaf senescence. Levels of two senescence markers on the days indicated were determined relative to those before ACC or MeJA treatment (Student’s *t* test, *P<0.05 and **P<0.01). Error bars indicate the SD (*n*=6).

In artificially induced leaf senescence, the genetic relationship between *ORE1* and *AtNAP* was further evaluated. Leaf senescence phenotypes were examined in wild-type, *ore1*, *atnap*, and *ore1 atnap* mutants during dark incubation. The leaves from the wild-type plants lost 84.4% of their chlorophyll after 6 d of dark incubation (Fig. 6A, B). However, for the *ore1* and *atna*p single mutants, the chlorophyll content declined more slowly; even after 6 d, ~35% of the chlorophyll was retained (Fig. 6A, B). Measurement of the photochemical efficiency of PSII showed that, after 6 d of dark incubation, the leaves of *ore1* and *atnap* still maintained >55% of their initial PSII activity, whereas wild-type leaves had lost their PSII activity almost entirely (Fig. 6C). In the *ore1 atnap* double mutant, 62.4% of the chlorophyll and 78.3% of the photochemical efficiency was retained even after 6 d of dark incubation (Fig. 6B, C). The leaf senescence symptoms of wild-type, *ore1*, *atnap*, and *ore1 atnap* mutants were assessed after treatment with two senescence-accelerating hormones, the ethylene precursor ACC, and MeJA. Not surprisingly, the leaves of the *ore1 atnap* mutant retained >70% of their chlorophyll following treatment with these hormones at 5 d after incubation, while ~50% and 40% of the chlorophyll was retained in each of the single mutant leaves treated with ACC and MeJA, respectively (Fig. 6D, F). A similar pattern was observed when photochemical efficiency was measured (Fig. 6E, G). Taken together, the *ore1 atnap* double mutant exhibited stronger senescence phenotypes than either of the single mutants. These results demonstrate that ORE1 and AtNAP might function in part additively in the regulation of leaf senescence and suggest that the two NAC TFs acting downstream of EIN3 independently regulate leaf senescence, but partially compensate for each other’s function.

### ORE1 and AtNAP control common as well as differential NAC TF genes

**Fig. 7.**
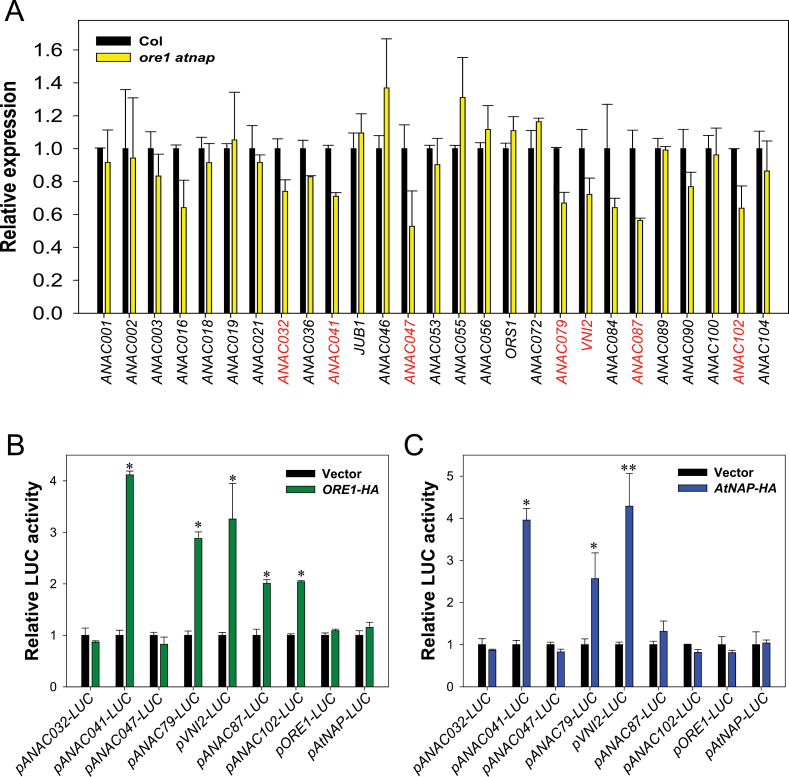
AtNAP and ORE1 control common as well as differential NAC TF genes. (A) Expression of 27 senescence-associated NAC TF genes in Col and *ore1 atnap* double mutant leaves at 16 d of leaf age. Transcript levels of each NAC TF gene were examined by qRT–PCR. *ACT2* was used as an internal control for qRT–PCR. Transcript levels of the NAC TF genes in the *ore1 atnap* mutant were determined relative to levels in wild-type leaves. The error bars represent the SD (*n*=4). The nine NAC TF genes whose expression was significantly decreased in the mature leaves of the *ore1 atnap* mutant, compared wirh levels in wild-type leaves, are highlighted by grey text. (B and C) Transactivation of the promoters of the selected NAC TF genes by ORE1 (B) and AtNAP (C) in *Arabidopsis* protoplasts. Protoplasts were co-transfected with each *NAC TF promoter-LUC* reporter and an effector plasmid expressing ORE1-HA or AtNAP-HA. Luciferase activity was determined relative to that in protoplasts that were transfected with the reporter plasmid and an effector plasmid expressing HA only. The relative expression of each *NAC TF promoter-LUC* was normalized to *35Sp:RLuc* (internal control) (Student’s *t*-test, *P<0.05 and **P<0.01). Error bars represent the SD (*n*=6).

It has been shown that the promoters of many NAC TF genes contain consensus NAC-binding sites (Olsen *et al.*, 2005; Balazadeh *et al.*, 2010). In addition, ANAC016 binds to the promoter of *AtNAP* and *ORS1* (Kim *et al.*, 2013). Moreover, a recent global gene expression analysis in the *ore1* mutant and inducible transgenic lines overexpressing *ORE1* has revealed that expression of several NAC TF genes is affected by ORE1 (Balazadeh *et al.*, 2010). Among them, *ANAC041* and *VNI2* have been predicted as direct targets of ORE1, because the promoters of the two genes have the ORE1 core binding site. These previous observations strongly support the possibility that ORE1 and AtNAP control leaf senescence, at least in part, through a NAC TF-mediated gene regulatory network. Therefore, qRT–PCR-based gene expression analysis was employed to identify the NAC TFs that lie downstream of ORE1 and AtNAP. Transcript levels of 27 senescence-associated NAC TF genes were evaluated in 16-day-old wild-type and *ore1 atnap* leaves (Fig. 7A). The expression of nine NAC TF genes was significantly reduced in *ore1 atnap* mutants, compared with wild-type leaves (Fig. 7A). Luciferase-based transactivation assays were performed using *Arabidopsis* protoplasts to determine the effect of ORE1 or AtNAP on the expression of seven NAC TF genes (Fig. 7B, C). The luciferase activity driven by the promoters of *ANAC041*, *ANAC079*, and *VNI2* was increased at least 2-fold by transiently overexpressed ORE1 and AtNAP. In contrast, the luciferase activity of *pANAC087-LUC* and *pANAC102-LUC* was only increased by overexpression of ORE1-HA, not AtNAP-HA. Interestingly, neither ORE1 nor AtNAP activated their own promoters or each other’s promoters. Taken together, these results suggest that ORE1 and AtNAP serve as key regulators of leaf senescence by controlling common and differential downstream NAC TFs.

## Discussion

In a previous study, a gene regulatory network was proposed as underlying leaf senescence, the trifurcate feed-forward pathway which involves EIN2, *miR164*, and ORE1 (Kim *et al.*, 2009). As a leaf ages, EIN2-mediated senescence signals induce the expression of ORE1, a positive regulator of leaf senescence, and simultaneously suppress the expression of *miR164*, which negatively regulates *ORE1* at the post-transcriptional level. Mathematical modelling and genetic analysis results further suggested the existence of an ORE1-independent pathway activated by EIN2-mediated senescence signals. Recently, EIN3 has been shown to be involved in the trifurcate feed-forward pathway, by directly repressing *miR164* expression (Li *et al.*, 2013). However, of what the gene regulatory network activated by EIN2-mediated senescence signal is composed and how it manages the leaf senescence process remains to be elucidated.

**Fig. 8.**
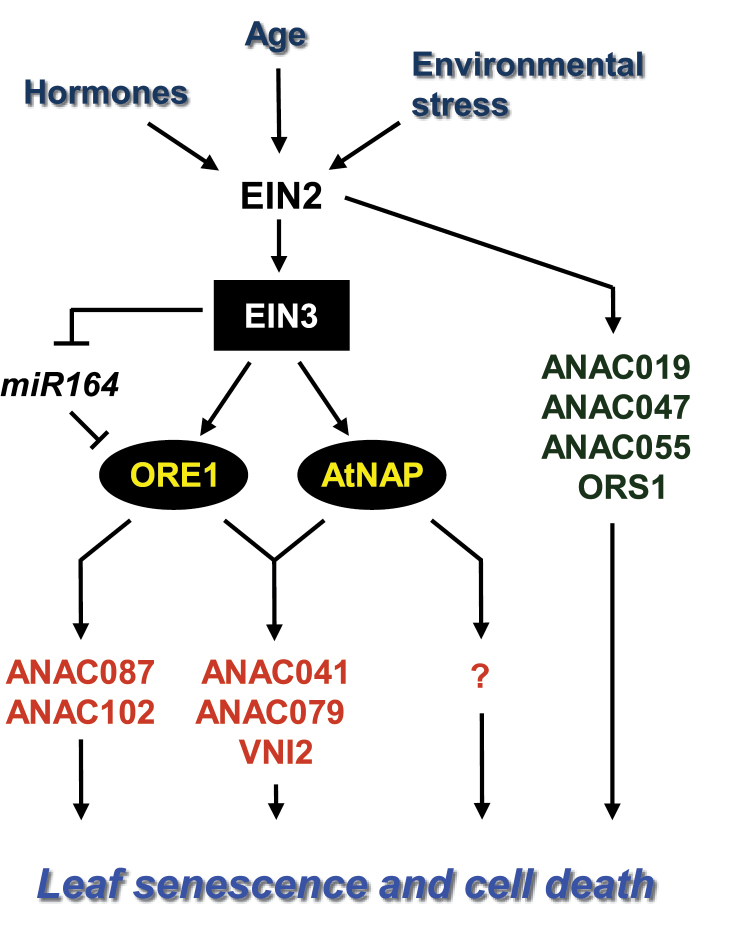
A plausible model for the EIN2–EIN3–NAC TFs regulatory cascade in the control of leaf senescence. EIN2-mediated senescence signalling, triggered by various senescence-inducing factors including age, hormones, and environmental stresses, activates EIN3. EIN3 directly induces the expression of two key positive regulators of leaf senescence, *ORE1* and *AtNAP*. Simultaneously, EIN3 directly suppresses the expression of *miR164* (Li *et al.*, 2013), which negatively regulates *ORE1* at the post-transcriptional level. ORE1 and AtNAP activate the expression of common as well as distinct downstream NAC TF genes. In addition, EIN2-mediated senescence signal is transduced to four NAC TFs (ANAC019, ANAC047, ANAC055, and *ORS1*) via an EIN3-independent pathway.

In this study, new molecular components of the trifurcate feed-forward pathway have been identified and characterized, enhancing understanding of the gene regulatory networks governing the leaf senescence process. New findings on the organization and function of the gene regulatory networks underlying leaf senescence included the following. First, six NAC TFs, including the two master regulators of leaf senescence, ORE1 and AtNAP, were found to participate in the gene regulatory network controlled by EIN2 (Fig. 1). Secondly, it was uncovered that EIN2-mediated senescence signal was transduced into ORE1 and AtNAP through the action of EIN3 (Figs 2–4). Furthermore, the data suggest that four additional NAC TFs seem to be mainly regulated by an EIN3-independent pathway(s) (Fig. 2). Thirdly, analysis of the downstream targets of ORE1 and AtNAP also provides new insights into how EIN2-mediated senescence signalling is differentially propagated through the key NAC TFs to execute the leaf senescence process (Fig. 7). Based on these data, a working model for an EIN2–EIN3–NAC TFs regulatory cascade with an important role in the control of leaf senescence was proposed (Fig. 8).

### NAC TFs as new components of the gene regulatory network activated by EIN2

EIN2, a central signalling component required for ethylene responses, has been long known as a master positive regulator of leaf senescence (Oh *et al.*, 1997; Alonso *et al.*, 1999), yet the gene regulatory network controlled by EIN2-mediated senescence signal is not fully understood. Here, an attempt was made to identify additional molecular components that act downstream of EIN2, as a first step towards understanding in detail the gene regulatory network activated by the EIN2-mediated senescence signal. In the present study, six NAC TF genes, including *ORE1*, *AtNAP*, and *ANAC055*, were found to be controlled by *EIN2*, based on gene expression analysis in the *ein2* mutant (Fig. 1). It is intriguing that no significant reduction in the transcript levels of the six NAC TF genes was observed in mature leaves of the *ore9* and *ore12* mutants (Fig. 1A). These data further support the possibility that EIN2 is one of the key regulators controlling the expression of NAC TFs during leaf ageing. It is also likely that EIN2 might not be the only route capable of activating the six NAC TF genes because transcript levels of all six NAC TFs were eventually increased as a leaf gets older (Fig. 1B–G). This is further supported by the previous finding that ANAC016, which directly binds to the promoter of *AtNAP* in yeast (Kim *et al.*, 2013), does not appear to be under the control of EIN2, based on the expression of *ANAC016* in the *ein2* mutant (Fig. 1A). Other TFs, including members of several TF families such as bZIP, bHLH, MYB, and AP2/ERF, were also identified as upstream molecules of ANAC019, ANAC055, and ANAC072 (Hickman *et al.*, 2013), indicating the complexity of the gene regulatory network involving NAC TFs. Thus, further experiments for identifying EIN2-independent senescence signalling pathways will be needed to better understand the gene regulatory networks involving NAC TFs in the control of leaf senescence. Interestingly, levels of some NAC TF transcripts, including *JUNGBRUNNEN1/ANAC042* reported as a negative regulator of leaf senescence (Wu *et al.*, 2012), were even higher in the *ein2* mutant. In the case of *ANAC055*, its transcript level was strongly reduced in the *ein2* mutant but significantly increased in the *ore9* and *ore12* mutants (Fig. 1). This result indicates that gene regulatory networks involving NAC TFs might be complex and interconnected. Elucidation of components acting downstream of EIN2 through genome-wide screening will help to characterize the complex global gene regulatory network activated by EIN2 to control leaf senescence.

### EIN3 directly activates the expression of *ORE1* and *AtNAP*

In this study, several lines of evidence support the conclusion that EIN3 is an upstream TF controlling the expression of *ORE1* and *AtNAP*. First, the expression of *ORE1* and *AtNAP* was significantly altered in *ein3 eil1* double mutants, even in young leaves (Fig. 2). Secondly, the *ore1* and *atnap* mutations partially suppressed the EIN3-induced early leaf senescence phenotype (Fig. 3). Thirdly, EIN3 directly bound to the promoters of the *ORE1* and *AtNAP* genes in Y1H and ChIP-PCR assays (Fig. 4A–C). Finally, transiently overexpressed *EIN3* was sufficient to activate the expression of *ORE1* and *AtNAP* (Fig. 4D).

It was notable that EIN3 functions as a transcriptional activator of *ORE1* and *AtNAP*, which are known to be positive regulators of leaf senescence (Oh *et al.*, 1997; Guo and Gan, 2006; Kim *et al.*, 2009), whereas EIN3 is known to function as a direct repressor of *miR164* (Li *et al.*, 2013). This suggests that EIN3 can function as both a transcriptional activator and a repressor, depending on the target genes to regulate leaf senescence, in agreement with a previous study (Shi *et al.*, 2012). This result further implies that EIN3 might simultaneously control the expression of *ORE1* and its negative regulator, *miR164*, to regulate leaf senescence efficiently.

Chang *et al.* (2013) revealed that EIN3 binds to the promoters of the four NAC TF genes (*ANAC019*, *ANAC047*, *ANAC055*, and *ORS1*) after ethylene treatment in *Arabidopsis* seedlings, but the expression of these NAC genes was not altered in the mature leaves of the *ein3 eil1* double mutant (Fig. 2B). This discrepancy implies that EIN3 and EIL1 might regulate different downstream targets under different physiological conditions or at different developmental stages. Thus, it is likely that the four NAC TF genes may not be direct targets of EIN3 and/or EIL1 in a mature leaf to trigger leaf senescence. Alternatively, it is equally possible that the four NAC TFs are targets of EIN3 and/or EIL1, but are regulated primarily by other TF(s) that act downstream of EIN2 in a mature leaf. In other words, the EIN2-mediated senescence signal seems to modulate leaf senescence through at least two independent pathways. These results imply that there are many routes to ensure leaf senescence and the associated cell death upon ageing. Future challenges will include identifying additional TFs that act downstream of the EIN2-mediated senescence signal, and determination of their molecular functions within the context of leaf senescence control.

### The effects of mutations in ORE1 and AtNAP are partially additive in the regulation of leaf senescence

The functional relationship between ORE1 and AtNAP that act downstream of EIN3 was also investigated by analysing the leaf senescence phenotype of the *ore1 atnap* double mutant (Figs 5, 6). The partially additive phenotypes of the *ore1 atnap* double mutant imply that ORE1 and AtNAP have both overlapping and independent functions in the transmission of EIN3-mediated senescence signals. This finding suggests that EIN3-mediated senescence signals can be transmitted via two partially independent pathways, one involving ORE1, and a second involving AtNAP.

Further understanding of how the gene regulatory network involving ORE1 and AtNAP functions in the regulation of leaf senescence can be facilitated by the identification of downstream target genes. Recent studies utilizing microarray analysis in the *ore1* mutant as well as inducible transgenic lines overexpressing *ORE1* have identified potential targets of ORE1. Among them, *BIFUNCTIONAL NUCLEASE1* has been characterized as a direct downstream target molecule of ORE1 (Balazadeh *et al.*, 2010; Matallana-Ramirez *et al.*, 2013). As a direct downstream target gene of AtNAP, *SAG113*, a gene encoding a protein phosphatase 2C family protein phosphatase, has been identified (Zhang and Gan, 2012). However, knowledge about the downstream gene regulatory network of ORE1 and AtNAP remains limited. In this study, downstream NAC TF genes of ORE1 and AtNAP were thus examined by performing transient transactivation assays in order to gain deeper insight into the gene regulatory networks involving ORE1 and AtNAP. It is intriguing that ORE1 and AtNAP did not activate each other, and *ANAC087* and *ANAC102* were preferentially activated by ORE1, not by AtNAP (Fig. 7B, C). The results further support the idea that ORE1 and AtNAP act independently in the regulation of leaf senescence by activating different NAC TFs, and they may differentially activate other downstream targets as well. ORE1 and AtNAP activated three common NAC TFs genes (*ANAC041*, *ANAC079*, and *VNI2*) (Fig. 7B, C), indicating that ORE1 and AtNAP might compensate for each other’s function by activating common downstream components.

It was notable that some of the NAC TFs activated by ORE1 in the transactivation assays were predicted as potential downstream targets of ORE1 in previous studies. For example, *ANAC102* is also one of the downstream genes predicted to be activated by ORE1 in a recent network modelling analysis based on high-resolution time-course profiles of gene expression during leaf development (Breeze *et al.*, 2011). It has also been known that *ANAC041* and *VNI2* might be downstream targets of ORE1 from a microarray analysis during leaf senescence (Balazadeh *et al.*, 2010; Rauf *et al.*, 2013). It is intriguing that ORE1 and AtNAP activate VNI2, a negative regulator of leaf senescence (Yang *et al.*, 2011). These findings imply that ORE1 and AtNAP, in addition to acting as positive regulators of senescence-accelerating genes, may finely tune the progression rate of leaf senescence through activating genes involved in maintenance activity. Further identification of direct downstream targets of ORE1 and AtNAP is essential to provide new insights into how the gene regulatory network involving the EIN2-mediated senescence signal regulates the leaf senescence process. Further experiments combining genetic analysis, ChIP-seq, gene expression profiling, and computational analyses will contribute to the elucidation of complex gene regulatory networks involving EIN2, EIN3, and NAC TFs, and will help us to understand how these pathways are interconnected. Collectively, the present data provide insight into the global gene regulatory network involving EIN3 and NAC TFs, through which the EIN2-mediated senescence signalling pathway coordinates global gene expression during leaf senescence.

## Supplementary data

Supplementary data are available at JXB online.

Table S1. Primers used in this work.

Supplementary Data
