# Supplementary material for: Gene regulatory cascade of senescence-associated NAC transcription factors activated by ETHYLENE-INSENSITIVE2-mediated leaf senescence signalling in Arabidopsis
Source: J Exp Bot. 2014 Mar 22;65(14):4023–36. doi: 10.1093/jxb/eru112 (PMC4106440; doi:10.1093/jxb/eru112)
Supplement: Supplementary Data [file supp_65_14_4023__index.html]

Gene regulatory cascade of senescence-associated NAC transcription factors activated by ETHYLENE-INSENSITIVE2-mediated leaf senescence signalling in Arabidopsis — Gene regulatory cascade of senescence-associated NAC transcription factors activated by ETHYLENE-INSENSITIVE2-mediated leaf senescence signalling in Arabidopsis — Supplementary Data 

# Gene regulatory cascade of senescence-associated NAC transcription factors activated by ETHYLENE-INSENSITIVE2-mediated leaf senescence signalling in *Arabidopsis*

## Supplementary Data

Data files

**Files in this Data Supplement:**

- Supplementary Data - Supplementary Data
