# Supplementary material for: Gene regulatory cascade of senescence-associated NAC transcription factors activated by ETHYLENE-INSENSITIVE2-mediated leaf senescence signalling in Arabidopsis
Source: J Exp Bot. 2014 Mar 22;65(14):4023–36. doi: 10.1093/jxb/eru112 (PMC4106440; doi:10.1093/jxb/eru112)
Supplement: Supplementary Data [file supp_eru112_jexbot116400_file001.pdf]

Supplemental Table S1. Primers used in this work.

Primer name	Sequence (5' to 3')	Purpose
pANAC003-F(SpeI)	GGACTAGTGGCTCACACAATTTACCCAACTTAC	Cloning
pANAC003-R(StuI)	GAAGGCCTTTCTCTGCAGCGATCAAAAAAG	
AtNAP-F(PstI)	AACTGCAGGATCGTAAATAATCTAAAGTTTGTGC	Cloning
AtNAP-R(StuI)	AGAGGCCTGATTTTCAGACAATTTAGAAAACAAT	
AtNAP-F(xmaI)	AACCCGGGGATCGTAAATAATCTAAAGTTTGTGC	Cloning
AtNAP-R(XhoI)	AGCTCGAGGATTTTCAGACAATTTAGAAAACAAT	
pANAC041-F(SpeI)	GGACTAGTGACAAGAGACATGACCTTTACCTTC	Cloning
pANAC041-R(StuI)	AGAGGCCTTAGAAAGAGAGACGAAAGAAGATGAG	
pANAC079-F(PstI)	AACTGCAGTATTAGACGACCAAATCTTATTGG	Cloning
pANAC079-R(StuI)	GAAGGCCTCTGTTTTTAAATAACCTTAAACCCTA	
pANAC083-F(SpeI)	GGACTAGTCGACAATAACGTCACGCCTTT	Cloning
pANCA083-R(StuI)	AGAGGCCTGGTGGTTCCAAACAAAGAGAGA	
pANAC087-F(PstI)	AACTGCAGCTTAATTTCGGAATTTACAGATACTTTG	Cloning
pANAC087-R(StuI)	GAAGGCCTCACTTTATATGTTTTTCAAGTAATAAAGATTC	
pORE1-F(PstI)	AACTGCAGAGATCGATTGTGTACACGAAGC	Cloning
pORE1-R(StuI)	AGAGGCCTTTTATCCTAATAGGGTTTCTAAAAATG	
pORE1-F(xmaI)	AACCCGGGAGATCGATTGTGTACACGAAGC	Cloning
pORE1-R(xhoI)	AGCTCGAGTTTATCCTAATAGGGTTTCTAAAAATG	
pANAC102-F(SpeI)	GGACTAGTGGAGCTTTTATAAACGAAGGGG	Cloning
pANAC102-R(StuI)	AGAGGCCTCTTCTCCTTCTCTTCCTCTCTGATG	
EIN3-F(BamHI)	GGATCCATGATGTTTAATGAGATGGGAATGTG	Cloning
EIN3-R(StuI)	AGGCCTGAACCATATGGATACATCTTGCTGC	
pmiRNA164A-F	ATGGTACGCCTAGCAACCTAGC	ChIP-qPCR
pmiRNA164A-R	CTTGTCTAATAACTGCTAAGT	
ACT2-F	GCGACTTGACAGAGAAGAAC	ChIP-qPCR
ACT2-R	GAA AGAGCGGAAGAAGATGAG	
AtNAPChIP-F1	GTCATGCAAACTATATCGTGGAG	ChIP-qPCR
AtNAPChIP-R1	GTTCTTCATTTCGCTATTATTGC	
AtNAPChIP-F2	CACGTGTTTAGAGGTGAAGAAC	ChIP-qPCR
AtNAPChIP-R2	GTCAAATGGTCATCAAACAGCC	
ORE1ChIP-F1	CCTCGTATGAACAAAACACGTG	ChIP-qPCR
ORE1ChIP-R1	GGTAATGATGATGAATGATGAG	
ORE1ChIP-F2	CAACACCAATAATAGTGACCCG	ChIP-qPCR
ORE1ChIP-R2	TGGTACTAATACGTAAGGTTAC	
ANAC001-F	CAGCAGAGCAAAGAAAAGGTG	qRT-PCR
ANAC001-R	TTGGTGGATGGAGGTATCTTG	
ANAC002-F	GATCATGCACGAGTACCGTCT	qRT-PCR
ANAC002-R	TGTAAATCCGGCAGAGAACC	

ANAC003-F	CCCTCAGCTTGTCAACAATTC	qRT-PCR
ANAC003-R	CATCCTCGATTGGAGCTTCT	
ANAC016-F	ATTCACTTCACAGTCAACAGGTG	qRT-PCR
ANAC016-R	GCTGATGAGAACTGGCTCCT	
ANAC018-F	AGAGTACAGATTCTTCCGGTGGT	qRT-PCR
ANAC018-R	GGAATCCTGGAGGGAGGTT	
ANAC019-F	AACTGTGGCTACCTGAAGACG	qRT-PCR
ANAC019-R	CCGAGTTATTAAACCCGTGACT	
ANAC021-F	TCTCTGAGCTCTCCAAAGGAA	qRT-PCR
ANAC021-R	AACAGCTTCCCATGTTGTCTC	
AtNAP-F	GAAACCAGACCATGTCTAAACCA	qRT-PCR
AtNAP-R	TTTCTCCAAACTCTGTTTTCTCG	
ANAC032-F	CAACAATTGGGCCTTAGCC	qRT-PCR
ANAC032-R	TGCTTCCTGAAAATAACAACACA	
ANAC036-F	GCTCAAGAAGACGCTTGTGTT	qRT-PCR
ANAC036-R	CTCGTTCATCACCCAATCAG	
ANAC041-F	AGGTGGAGAATGAGAAAGAGACA	qRT-PCR
ANAC041-R	TCTCATAAAGTCATAAAATATGGGACA	
ANAC042-F	ACCAAAACCGATTGGATGAT	qRT-PCR
ANAC042-R	TTCTGCAAAGTGTCCATACCTC	
ANAC046-F	GAGCCATGGGATCTACCAAA	qRT-PCR
ANAC046-R	GATCCCTCTGGCAGAAAAAGT	
ANAC047-F	AGCGGTGGTAGCGAACTG	qRT-PCR
ANAC047-R	TGATGACAATGAAGCGTGTG	
ANAC053-F	GCAACAGAGTTTGAGCCAGA	qRT-PCR
ANAC053-R	GCAGGAATAGCACCCAACAT	
ANAC055-F	TTCTCGAGTCGTTGCATGAG	qRT-PCR
ANAC055-R	CTATGAGGCAGCGCGTTT	
ANAC056-F	CGAAAACAAACCAAACAATCG	qRT-PCR
ANAC056-R	TTCTACATAACACCCAATCATCAAGT	
ANAC059-F	AGTAAAAACAAATTGGGTCATGC	qRT-PCR
ANAC059-R	AAAAACACGACTAATAACACATTCGT	
ANAC072-F	GCACGAGTATCGCTTAATAGAACA	qRT-PCR
ANAC072-R	CGACACAACACCCAATCATC	
ANAC079-F	AACTTGCCTAAAACCGCTAAGA	qRT-PCR
ANAC079-R	AGAACCGATTCGGATTAACG	
ANAC083-F	AAGAACCGATTGGATCATGC	qRT-PCR
ANAC083-R	AGTGGGACCCATAGAACTCG	
ANAC084-F	GATTTGCGTCGGCTAAGGT	qRT-PCR
ANAC084-R	TTCTTCATCATCTCCCTCTCTTG	

ANAC087-F	AGTGTGAGCCTTGGGATTTG	qRT-PCR
ANAC087-R	CCCAGTCGGATACTTCCTGT	
ANAC089-F	GGGGAGAAACTACTCGCTGA	qRT-PCR
ANAC089-R	CGCAAAGAAATCCTCGTCA	
ANAC090-F	CGAGCCTAGTCATCTTCCAAA	qRT-PCR
ANAC090-R	AGAACCATTGCTCAGCGTCT	
ORE1-F	GTGGGTATGAAGAAAACTTTGG	qRT-PCR
ORE1-R	TTCGTTCTTAGCTGTTTGGGG	
ANAC100-F	TGAGTACAGGCTTGAAGGAAAA	qRT-PCR
ANAC100-R	CCTGCATATCACCCATTCATT	
ANAC104-F	ATTACGATCCTTGGGACCTTC	qRT-PCR
ANAC104-R	TGCTTGTCACTCTCTCTTGTGTC	
gORE1-F	GTCTTTTGTCTTCGGTTTCT	Genotyping
gORE1-R	TGTGAGAGACCGGAAATACC	
